# The Documentation of Goals of Care Discussions at a Canadian Academic Hospital

**DOI:** 10.7759/cureus.9560

**Published:** 2020-08-04

**Authors:** Jaime-Lee Munroe, Stuart L Douglas, Timothy Chaplin

**Affiliations:** 1 Family Medicine, Queen's University, Kingston, CAN; 2 Emergency Medicine, Queen's University, Kingston, CAN

**Keywords:** goals of care, advance care planning, cardiopulmonary resuscitation, end of life care, quality of life, advance directives, cardiac arrest, medical record, do not resuscitate orders, patient-centred care

## Abstract

Introduction: Patient-centered care is a core principle of the Canadian healthcare system. In order to facilitate patient-centered care, the documentation of a patient’s medical goals and expectations is important, especially in the event of acute decompensation when an informed conversation with the patient may not be possible. The ‘Goals of Care Discussion Form (GCF)’ at Kingston Health Sciences Centre (KHSC) documents goals of care discussions between patients and healthcare providers. All patients admitted to the Internal Medicine service are expected to have this form completed within 24 hours of admission. Formal measurement of form completion at our center has not previously been done, though anecdotally this form is often incomplete. The purpose of this study is to quantify the rate of completion and assess quality of documentation of the GCF at KHSC.

Methods: This prospective chart review took place between August 25, 2018, and March 25, 2019. Charts were reviewed for the presence of a completed GCF, and the quality of notation was assessed, as appropriate. Given there are no existing tools for assessing the quality of a document such as the GCF, authors TC and JM created one de novo for this study. Extracted data included the amount of time elapsed between admission and completion of the GCF, whether the ‘yes/no cardiopulmonary resuscitation (CPR)’ order in the patient’s chart aligned with their wishes as outlined on the GCF, and whether or not a patient’s GCF was uploaded to the hospital’s electronic medical record (EMR).

Results: Two hundred sixteen charts were reviewed. Of these, 136 (63.0%) had a complete GCF. The mean GCF quality score was 3.4/7 (95% CI [3.2, 3.6]). The mean time elapsed from admission to the completion of the GCF was 1.5 days (95% CI [0.6, 2.4]). There were 130 charts with both a complete GCF and a ‘yes/no CPR’ order, and of these, 20 (15.4%) showed a discrepancy. Eighty-six (63.2%) of the completed GCFs were uploaded to the EMR.

Discussion and conclusions: The rate of GCF completion at KHSC is noticeably higher than expected based on the previous literature. However, our assessment of the quality of completion indicates that there is room for improvement. Most concerning, discrepancies were found between the ‘yes/no CPR’ order in a patient’s chart and their stated wishes on the GCF. Furthermore, less than two-thirds of completed GCFs were found to have been uploaded to the hospital’s EMR. Given the emphasis on patient-centered care in the Canadian healthcare system, our findings suggest that improvement initiatives are needed with respect to documenting goals of care discussions with patients.

## Introduction

Patient-centered care is a core value of the Canadian healthcare system, and is predicated upon a patient having an understanding of his or her disease process, prognosis, and the benefits and risks of treatment options [1-3]. In order to deliver patient-centered care, medical teams must ensure their patients are well-informed, and must seek to understand and respect their patients’ values and wishes [2,3].

The goal of advance-care planning is to engage in and document discussions with patients while they are capable [4,5]. This facilitates patient-centered care in the case of acute decompensation, when a patient may not be capable of making an informed decision [6,7]. Failure to properly document these discussions and decisions can lead to adverse outcomes both for patients and healthcare providers [6,8]. These outcomes include the unnecessary use of resources, medico-legal consequences, and more importantly, the provision of unwanted interventions that may lead to unnecessary physical and emotional harm to patients, their families, and healthcare providers [6,8].

Keeping the above-mentioned factors in mind, Kingston Health Sciences Centre (KHSC) has implemented an advance-care planning document called a ‘Goals of Care Discussion Form’ (GCF). An example of the GCF has been included under the 'Discussion' section of this article. The GCF is a bright blue, two-sided document that resides in the paper patient chart, and its purpose is to facilitate and document a goals of care discussion. Side 1 documents the individuals present for the discussion, as well as the discussion content and outcome. Side 2 provides guidance for the individual completing the GCF on how to prepare for, hold, and follow up after the goals of care discussion. The GCF is not a medical order but is often used to inform a patient’s resuscitation status. It is completed by a member of the patient's healthcare team, with an informal target of completion within 24 hours of admission to the Internal Medicine service at KHSC. Despite endorsement from hospital leaders and administrators, it is unknown if the GCF is actually being completed within this timeframe.

The purpose of this study is to assess the rate and quality of completion of the GCF at the Internal Medicine service at KHSC. Secondary outcomes of interest include the rate of agreement between patients' wishes for cardiopulmonary resuscitation (CPR) as outlined on their GCF and the ‘yes/no’ CPR order in their chart, the time elapsed between admission to hospital and completion of a GCF, and the number of complete GCFs uploaded to the electronic medical record (EMR).

## Materials and methods

This was a prospective chart review study of patients currently admitted to the Internal Medicine service at the Kingston General Hospital (KGH) site of KHSC on data collection days. The Internal Medicine service has approximately 150 inpatients at any given time. A convenience sample of 250 charts was decided *a priori*; this number was informed by similar studies where sample sizes varied from approximately 150 to 3400 charts [5,7-10]. Data collection took place on six days, based on the availability of author JM, over a six-month study period. This period was chosen in an attempt to minimize any variation in GCF completion that may have been attributed to individual physicians or learners, who typically rotate through the Internal Medicine service for periods of two to six weeks. Efforts were also made to ensure adequate time between data collection days to limit the chances of encountering individual charts multiple times for those patients with weeks- or months-long hospital admissions. Data was abstracted using a standardized spreadsheet.

Analysis plan

Rate of GCF Completion

A GCF was considered ‘complete’ when it was found in a patient’s paper chart with the ‘signature, date, and time’ section completed, and there was information recorded under any one of the three main sections (Part I, Part II, or Part III).

GCF Quality Score

**Figure 1:**
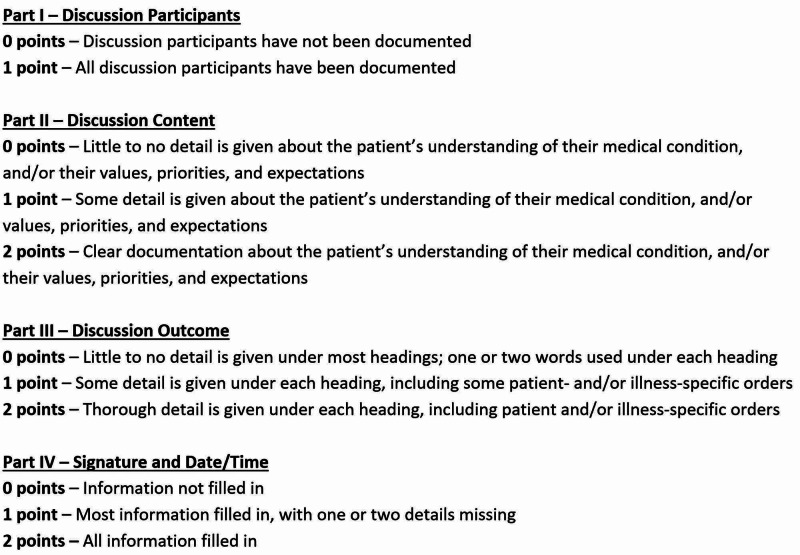
The GCF scoring tool GCF, Goals of Care Discussion Form

As there are no existing tools that assess the quality of completion of goals of care documents, one was created for this study (Figure 1). It was developed by study authors (TC and JM) with input from a palliative care physician.

The quality assessment tool was designed to be pragmatic and simple to use. It assigns a numerical score to each section of the GCF based on the level of detail provided, and whether this information appeared to be generic, or patient-specific. GCFs were then given a total ‘quality score’ out of 7. In the ‘Discussion Participants’ section (i.e., Part I), a score of 0 was assigned if discussion participants had not been documented, and a score of 1 was assigned if discussion participants were documented. The remaining sections were scored from 0 to 2. A score of 0 was assigned when ‘little to no detail’ was recorded, with responses that were often one to two words long. A score of 1 was given for ‘some detail’, with some patient- and/or illness-specific information, while a score of 2 was assigned when there was clear and thorough documentation of patient-specific information. Study authors, JM and TC, discussed and reviewed the scoring rubric to ensure a shared understanding of the quality score prior to data collection.

It is important to note that this scoring tool was used only to assess the quality of GCF documentation. Study authors were not present for and did not assess the goals of care discussions themselves, nor were they privy to the content of these discussions beyond what was provided in writing on the GCF.

All charts were assessed and scored by the primary author (JM). Intra-rater reliability (IaRR) of the scoring tool was assessed when the primary author encountered the same GCFs on multiple data collection days. A second author (TC) independently scored a random sample of 20 charts to assess inter-rater reliability (IeRR).

Time Between Patient Admission Date and GCF Completion Date

Patient admission date was determined using the standard demographics sheet in the patient chart. GCFs are signed and dated upon completion. This information was used to calculate the mean time (in days) between patient admission and GCF completion.

‘Yes/No CPR’ Order and the GCF Discussion Outcome

Patients’ ‘yes/no CPR’ admission orders were compared against the corresponding section on their GCF.

Scanning of GCFs to the EMR

A typical hospital practice is to have GCFs scanned and uploaded to the EMR system within two days of a patient’s discharge from hospital. The KHSC EMR was accessed at least 10 days after the initial chart review. Any GCF not present in the EMR at that time was considered to be ‘not uploaded’, including GCFs for patients who were currently admitted to the Internal Medicine service.

## Results

GCF completion rate and quality score

Two hundred sixty-two charts were reviewed over six data collection days between August 2018 and March 2019. Forty-six of these were excluded from analysis, as they were encountered on more than one data collection day due to their belonging to patients with weeks- or months-long hospital admissions. This left 216 unique charts for analysis. Of these, 136 (63.0%) charts had a complete GCF, and 80 (37.0%) had a GCF that was either incomplete or missing. The mean quality score for the completed GCFs was 3.4/7 (95% CI [3.2, 3.6]).

For 17 out of 20 GCFs, the scores given by reviewers matched (IeRR = 0.85), and those for the remaining three GCFs were within 1 point. In addition, among the 46 excluded charts, repeat GCFs were encountered 18 times by the primary investigator (JM) on separate data collection days, and scored blindly. In 15 of 18 encounters, the scores matched. In the remaining three encounters (IaRR = 0.83), scores for two of the charts were within 1 point, and for the remaining chart, the score was within 2 points.

Time elapsed between the patient admission date and GCF completion date

The mean time to completion of the GCF was 1.5 days (95% CI [0.6, 2.4]) from the day of patient admission. One hundred fifteen (84.6%) GCFs were completed within the 24-hour target. Twenty-one (15.4%) GCFs were delayed an average of 9.8 days before completion.

‘Yes/no CPR’ order and the GCF discussion outcome

There were 130 unique cases where both the patient’s code status as determined by the physician orders, and the patient’s wishes as outlined on the GCFs, could be ascertained. In 20 (15.4%) such cases, these documents did not match.

Scanning of GCFs to the EMR

One hundred thirty-six GCFs were searched for in KHSC’s EMR. Of these, 86 (63.2%) were uploaded to KHSC’s EMR.

## Discussion

**Figure 2:**
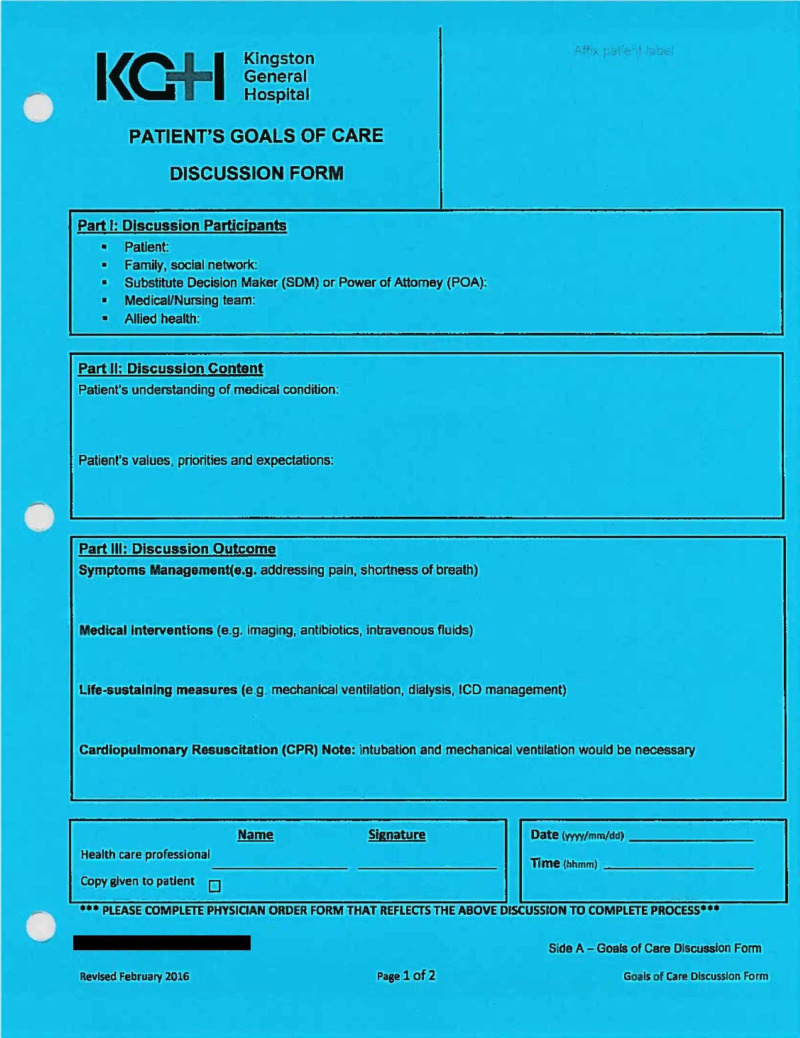
Side 1 of the GCF GCF, Goals of Care Discussion Form

**Figure 3:**
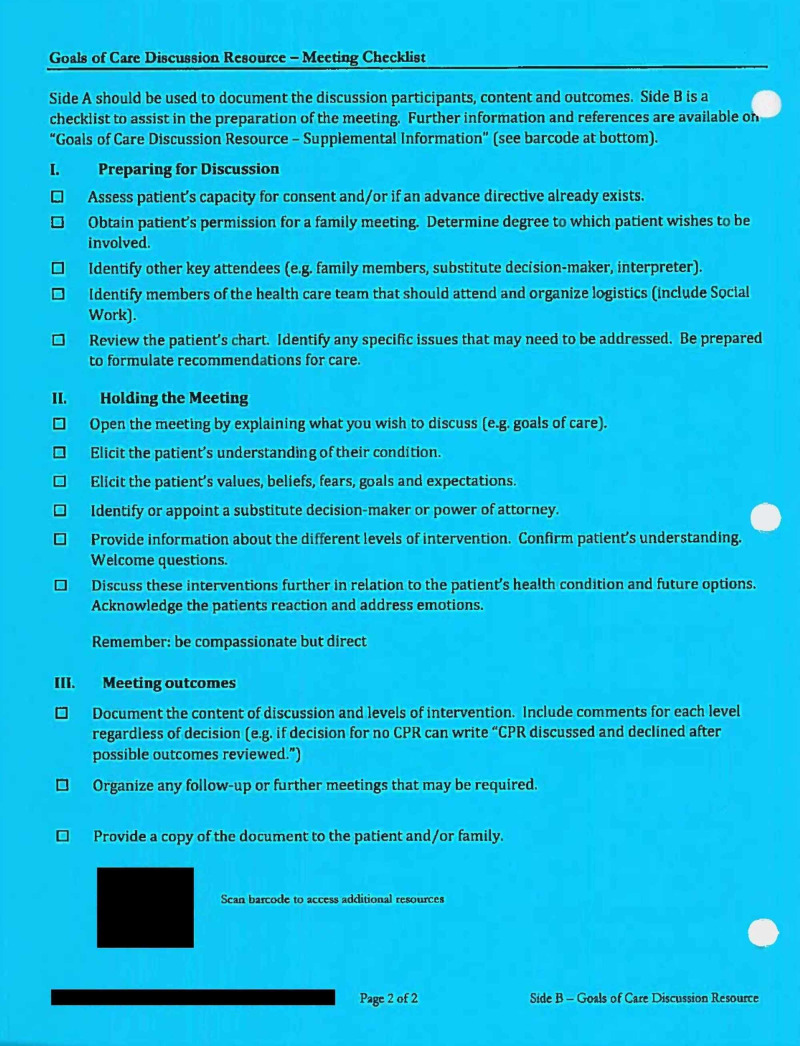
Side 2 of the GCF GCF, Goals of Care Discussion Form

The completion rate of GCFs in our chart review was 63.0%. This is noticeably higher than the completion rate of advanced goals of care documentation seen in similar studies, where completion rates ranged from 9.3% to 27.2% [5,7-10]. However, these results may not be directly comparable, as these studies primarily focused on the completion of a do-not-resuscitate (DNR) form. A DNR form is not typically institution specific, and is an official physician order that addresses a patient's wishes only with respect to CPR, while a GCF is institution specific, not an official physician order, and used to facilitate and document goals of care discussions regarding multiple aspects of patient care (Figures 2, 3).

While the completion rate of 63.0% is higher than expected, the quality of completion as determined by our scoring tool was limited. The average quality score was 3.4/7 (95% CI [3.2, 3.6]); a score of 2/7 could be earned by simply completing the ‘signature, date, and time’ section of the GCF. These results indicate an ongoing challenge with respect to the effective documentation of goals of care discussions. Although an assumption, if the quality of documentation reflects the quality of the conversation between the patient and physician, there is likely a larger role that education can play in improving the utility of these forms. If they are to accurately communicate a patient’s values and goals, then further efforts are required to improve the quality of documentation.

Another promising finding of the study was the short time interval between admission and GCF completion. In fact, 111 GCFs had a time elapsed to completion of 0 days (95% CI [0.6, 2.4]). This likely reflects institutional support for their timely documentation and affirms the importance of these forms.

Despite our finding that the majority of GCFs were completed at the time of admission orders, which include the ‘yes/no CPR’ order, these two documents did not align in 20 (15.4%) of 130 charts. This is concerning for multiple reasons. First, a discrepancy suggests that patients may not understand the questions being asked of them, or their answers are interpreted differently between healthcare providers (e.g., if different individuals complete the admission orders versus the GCF). Second, it creates confusion with respect to a patient’s ‘true’ wishes and raises the possibility that a patient could receive discordant care.

Timely and easy access to a patient’s GCF through the EMR can be very helpful as a starting point for discussions in case of an emergency department visit or readmission in the future. In our sample of charts, 63.2% of completed GCFs were uploaded to the EMR. Improving this rate is another area to focus quality improvement energy.

Limitations

There are multiple limitations to this study. First, this was a single-institution study and our findings may not reflect the rates of completion at other hospitals where local cultures and resources are different. Second, our use of convenience sampling for the chart review introduced the potential for selection bias. Third, for a portion of the charts, the EMR was accessed at least 10 days after chart review rather than specifically after patient discharge; this may have skewed the results regarding the scanning and uploading of the GCF to the EMR. Finally, our ‘quality scoring tool’ does not have previous evidence for its validity and the results should be interpreted with caution. Although we did design the tool to be as objective and pragmatic as possible, and we provide some evidence of intra- and inter-rater reliability, the ‘quality’ of a GCF is not easily defined with a simple number.

## Conclusions

Although the rate of completion of GCFs at KHSC was higher than expected, significant findings that could lead to care discrepant with patient values underscore the importance of this study. These include the discrepancies in the documentation of patient code status, and the GCF being absent from the hospital EMR in a number of cases. Future work will focus on local initiatives to improve the quality of goals of care discussions and their documentation in order to deliver patient-centered care. We suggest similar evaluation at other centers to guide local quality initiatives.
